# Unveiling the influence of lateralized carotid artery revascularization on cognitive function: a comparative analysis

**DOI:** 10.3389/fneur.2025.1631701

**Published:** 2025-12-05

**Authors:** Sensen Wu, Yachan Ning, Hui Wang, Julong Guo, Xixiang Gao, Chunmei Wang, Yongquan Gu, Lianrui Guo

**Affiliations:** 1 Department of Vascular Surgery, Xuanwu Hospital, Capital Medical University, Beijing, China; 2 Department of Intensive Care Medicine, Xuanwu Hospital, Capital Medical University, Beijing, China

**Keywords:** cognition, carotid artery revascularization, carotid endarterectomy, carotid artery stenting, stroke

## Abstract

**Objective:**

To investigate and compare the changes in cognitive function after carotid artery revascularization surgery on different sides.

**Methods:**

From April 2019 to April 2021, patients with ≥70% carotid artery stenosis who were treated with carotid endarterectomy (CEA) or carotid artery stenting (CAS) were recruited for this study. The Montreal Cognitive Assessment (MoCA) instrument was used to evaluate cognitive function preoperatively and at 3, 6, and 12 months postoperatively. Patients were divided into two groups based on the side of the surgery, repeated measures ANOVA was used for comparisons.

**Results:**

A total of 89 patients who met the criteria were enrolled and completed 1-year follow-up. At 3, 6, and 12 months after carotid revascularization, the total MoCA score and delayed recall score were significantly improved compared with the baseline scores (*p* < 0.05). In patients who underwent left-sided revascularization, verbal fluency showed improvement at 12 months compared to baseline levels (*p* < 0.05). In patients who underwent right-sided revascularization, attention showed improvement at 6 months compared to baseline, and the improvement in delayed recall at 6 and 12 months was still evident compared to the 3-month assessment (*p* < 0.05).

**Conclusion:**

Carotid revascularization can improve cognitive function in patients, with differences in cognitive function changes observed between left and right carotid revascularization procedures.

## Introduction

Cognitive function refers to an individual’s ability to receive, process, and apply information, encompassing aspects such as memory, learning, thinking, understanding, judgment, and problem-solving. Decline in cognitive function can result in impairment across various cognitive domains, including language barriers, decreased computational abilities, weakened judgment, declining memory, visual–spatial dysfunction, decreased executive function, among others, thereby impacting behavior, emotions, and personality. Ultimately, this reduction in cognitive function reduces work and daily life capabilities, imposing a significant economic burden and psychological stress on families and society (1, 2). Meanwhile, mild cognitive impairment (MCI) is closely linked to an increased risk of developing Alzheimer’s disease (AD). MCI is currently recognized as an early stage of dementia, with about 10 to 15% of MCI patients progressing to AD each year, a risk ten times higher than that of the normal population. Identifying and intervening in potential risk factors for MCI conversion to AD is crucial for preventing AD, and reducing the progression of cognitive decline during the MCI stage may be a key strategy in preventing its transformation into dementia (3).

Carotid artery stenosis is one of the most common causes of ischemic stroke, with a prevalence of severe atherosclerotic carotid artery stenosis (defined as stenosis ≥70%) ranging from approximately 0.1 to 3% in the general population. As age advances, notably in the age group of 65 years and older, the prevalence of carotid artery stenosis significantly rises. It is estimated that around 75% of men and 62% of women will experience varying degrees of carotid artery stenosis, with some individuals experiencing stenosis exceeding 50%. The prevalence of carotid artery stenosis is progressively increasing due to population aging and lifestyle alterations, with this trend continuing to escalate (4, 5). Besides acute cerebrovascular events, carotid artery stenosis can also lead to chronic cerebral hypoperfusion, resulting in cognitive decline. In the early 1950s, Fisher et al. (6) first suggested that carotid artery disease and impaired blood flow can impact cognitive function. Studies (7–9) have demonstrated a correlation between carotid artery stenosis and cognitive impairment, with the findings of the Tromsø study (10) indicating that subjects with carotid artery stenosis performed significantly lower on various cognitive tests.

Cervical artery revascularization is a treatment method aimed at effectively improving blood flow in the cervical arteries, alleviating symptoms of cerebral hypoperfusion, and preventing cerebrovascular accidents. Current evidence suggests that carotid endarterectomy (CEA) and carotid artery stenting (CAS) can prevent future strokes in patients with cervical artery stenosis (11, 12). However, consensus has not been reached regarding their impact on cognitive function. Additionally, given the inherent functional differences between the left and right cerebral hemispheres, it is theoretically plausible that patients with stenosis in different areas of the cervical arteries may experience varying effects on cognitive function. Previous studies (13–15) have indicated that patients undergoing left CEA surgery exhibited significant improvement in language function, while those undergoing right CEA demonstrated an improvement in executive function to some extent. Nevertheless, due to limited prior research and some studies yielding non-positive results (16–18), it is imperative to conduct independent studies on patients with stenosis on different sides to gain a better understanding of postoperative changes in cognitive function. Changes in cognitive function can have a significant impact on the quality of life, particularly for asymptomatic patients with cervical artery stenosis. Understanding the effects of cervical artery revascularization on cognitive function is crucial. This study aims to investigate the changes in cognitive function after cervical artery revascularization on different sides and compare the effects of left and right revascularization on cognitive function. The findings will provide new insights and strategies for the clinical treatment of cognitive impairments related to cervical artery stenosis, ultimately improving the quality of life for patients.

## Methods

### Study population and criteria

This study is a single-center prospective observational study that includes patients with severe carotid artery stenosis who underwent carotid endarterectomy or carotid artery stenting from April 2019 to April 2021 in our department of vascular surgery. The study has been approved by the hospital ethics committee, and all subjects have obtained informed consent from themselves or their legally authorized representatives.

Inclusion Criteria: (1) Age ≥ 40 years; (2) Patients with unilateral severe carotid artery stenosis (≥70%) scheduled for carotid endarterectomy or carotid artery stenting; (3) Able to independently complete relevant cognitive function assessment scales; (4) Patient’s informed consent.

Exclusion Criteria: (1) Patients who refuse to cooperate with cognitive function assessment or other related examinations; (2) Patients with a history of depression and mental illness; (3) Patients with known causes of cognitive impairment such as Parkinson’s disease, Alzheimer’s disease, epilepsy, or other cognitive impairment-related diseases; (4) Patients with aphasia, dysarthria, visual or hearing impairment; (5) Patients who have previously undergone carotid endarterectomy or carotid artery stenting; (6) Patients with severe systemic diseases or significant organ dysfunction; (7) Patients with malignant tumors; (8) Patients with an estimated lifespan of less than 1 year.

### Data collection and neurocognition assessment

Trained vascular surgeons will conduct detailed interviews with patients to collect medical history, perform physical examinations, and gather general information and baseline characteristics. This includes collecting general information such as age, gender, education level, hypertension, hyperlipidemia, diabetes, coronary artery disease, atrial fibrillation, smoking history, alcohol consumption history, cerebral white matter changes, lesion location, features of the plaque, and degree of stenosis. Additionally, relevant laboratory tests including complete blood count, liver and kidney function, lipid profile, enzyme spectrum, C-reactive protein, electrolytes, and coagulation function will be conducted. All eligible patients will undergo magnetic resonance imaging (MRI) and diffusion-weighted imaging (DWI) sequences before carotid artery revascularization and within one week postoperatively to determine the occurrence of subclinical microembolism.

According to the guidelines of the Chinese Interventional Stroke Registry system, this study will utilize the Chinese version of the Montreal Cognitive Assessment (MoCA) test to assess the cognitive function of enrolled patients (19, 20). The cognitive function tests will be conducted by trained and certified doctors in a quiet environment. The testing time points will be prior to surgery (within one week), at 3 months, 6 months, and 12 months postoperatively. The MoCA test assesses various cognitive domains, including visual–spatial skills, executive functions, naming, memory, attention, verbal fluency, abstraction, delayed recall, and orientation. The MoCA score ranges from 0 to 30, with higher scores indicating better cognitive abilities. Normal cognition is defined as a score of 26 or above. For patients with ≤12 years of education, one point will be added to their MoCA total score to correct for the bias caused by educational level.

### Carotid revascularization procedures and endpoints

All surgeries will be performed by experienced vascular surgeons. The choice of surgical technique for carotid artery revascularization will be determined by the vascular surgeons. Patients will receive aspirin (100 mg/day) for at least 3 days before CEA and a combination of aspirin (100 mg/day) and clopidogrel (75 mg/day) for at least 3 days before CAS. All patients will receive aspirin (100 mg/day) and clopidogrel (75 mg/day) for at least 3 months, followed by long-term use of a single antiplatelet drug.

The main endpoints of this study include MoCA assessment of cognitive function at 3 months, 6 months, and 12 months postoperatively. The occurrence of hemodynamic impairment and complications within one month postoperatively will be observed. Patients will undergo vascular ultrasound examinations at 3 months, 6 months, and 12 months postoperatively to observe the occurrence of carotid artery restenosis. All patients will be followed up during outpatient visits.

### Statistical analysis

Statistical analyses were performed using SPSS software (version 26.0, IBM Corp., Armonk, NY, United States) and R software (version 4.2.2; R Foundation for Statistical Computing, Vienna, Austria). Continuous variables conforming to a normal distribution are presented as mean ± standard deviation (SD), whereas non-normally distributed variables are presented as median (interquartile range). Categorical variables are expressed as counts and percentages. Comparisons between categorical variables were conducted using the chi-square test or Fisher’s exact test, as appropriate. Continuous variables were compared using the Welch’s two-sample t-test. For both within-group (pre- vs. postoperative) and between-group (left- vs. right-sided) comparisons across multiple time points, a repeated measures analysis of variance (ANOVA) was applied. The sphericity assumption was assessed by Mauchly’s W test; when violated, the Huynh–Feldt correction was employed. In cases where repeated measures ANOVA indicated statistically significant differences, Tukey’s Honestly Significant Difference (HSD) test was used for *post hoc* pairwise comparisons. A two-sided *p* value < 0.05 was considered statistically significant.

## Results

### Demographic and clinical characteristics of patients

A total of 97 patients were enrolled in this study, including 73 males (82.0%). The mean age was 66.9 ± 8.5 years. Among all participants, 44 patients (49.4%) presented with symptomatic carotid artery stenosis, defined as the occurrence of neurological symptoms within the previous six months. Most patients had at least one chronic comorbidity, including hypertension in 68 cases (76.4%), diabetes mellitus in 35 (39.3%), coronary artery disease in 28 (31.5%), and hyperlipidemia in 43 (48.3%). In addition, 44 patients (49.4%) demonstrated cerebral white matter lesions on MRI. Patients were divided into two groups according to the side of revascularization, with 39 patients in the left-sided group and 50 in the right-sided group. As summarized in Table 1, there were no statistically significant differences between the two groups in age, sex distribution, education level, degree of stenosis, or chronic comorbidities.

**Table 1:** Basic information of patients.

Characteristic	Total (*n* = 89)	Left (*n* = 39)	Right (*n* = 50)	*p*-value*
Demographic and clinical characteristics				
Age, year	66.94 ± 8.53	67.85 ± 9.97	66.24 ± 7.25	0.381
Mean ± SD				
Male, *n*(%)	73 (82.02)	31 (79.49)	42 (84.00)	0.582
Education, year	9.24 ± 3.75	9.59 ± 3.88	8.96 ± 3.66	0.435
Mean ± SD				
BMI Mean ± SD	24.97 ± 2.78	24.86 ± 2.64	25.06 ± 2.90	0.738
Symptomatic stenosis, *n*(%)	44 (49.44)	22 (56.41)	22 (44.00)	0.245
CAD, *n*(%)	28 (31.46)	14 (35.90)	14 (28.00)	0.426
Hypertension, *n*(%)	68 (76.40)	29 (74.36)	39 (78.00)	0.688
Diabetes, *n*(%)	35 (39.33)	15 (38.46)	20 (40.00)	0.883
Hyperlipidemia, *n*(%)	43 (48.31)	17 (43.59)	26 (52.00)	0.431
Smoking history, *n*(%)	45 (50.56)	22 (56.41)	23 (46.00)	0.330
Alcohol consumption, *n*(%)	36 (40.45)	20 (51.28)	16 (32.00)	0.066
Leukoencephalopathy, *n*(%)	44 (49.44)	17 (43.59)	27 (54.00)	0.330
Stenosis severity, *n*(%)				0.798
50–69%	4(4.49)	2(2.25)	2(2.25)	
70–99%	85(95.5)	37(41.57)	48(53.93)	
Ipsilateral vertebral artery stenosis, *n*(%)	79 (88.76)	35 (89.74)	44 (88.00)	1.000
Ulcerative plaques, *n*(%)	6 (6.74%)	4 (10.26%)	2 (4.00%)	0.398
Perioperative outcomes				
Surgical techniques, *n*(%)				0.559
CEA	54 (60.67)	25 (64.10)	29 (58.00)	
CAS	35 (39.33)	14 (35.90)	21 (42.00)	
Ministroke, *n*(%)	7 (7.87)	3 (7.69)	4 (8.00)	1.000
Hypotension, *n*(%)	4 (4.49)	2 (5.13)	2 (4.00)	1.000
Bradycardia, *n*(%)	7 (7.87)	3 (7.69)	4 (8.00)	1.000
Hematoma, *n*(%)	1 (1.12)	0 (0.00)	1 (2.00)	1.000
Nerve injury, *n*(%)	5 (5.62)	3 (7.69)	2 (4.00)	0.774
New infarcted lesions in MRI, *n*(%)	8 (8.99)	4 (10.26)	4 (8.00)	1.000

^*^Welch Two Sample *t*-test; Pearson’s Chi-squared test; Fisher’s exact test. BMI: Body mass index; CAD: Coronary artery disease; SD: standard deviation. CEA: carotid endarterectomy; CAS: carotid artery stenting; MRI: Magnetic resonance imaging.

### Postoperative outcomes within 30 days

Perioperative outcomes are summarized in Table 1. Among all patients, 60.7% underwent CEA, while the remaining patients received CAS. Seven patients (7.2%) experienced minor postoperative strokes, whereas no major strokes were reported. Postoperative hypotension occurred in four patients (4.1%), all of whom had undergone CAS. Additionally, five patients (5.2%) developed cranial nerve injuries. Follow-up magnetic resonance imaging (MRI) performed within 7 days after surgery identified new small ischemic infarctions in eight patients (8.2%).

### Effect of revascularization on cognition among all patients

**Figure 1:**
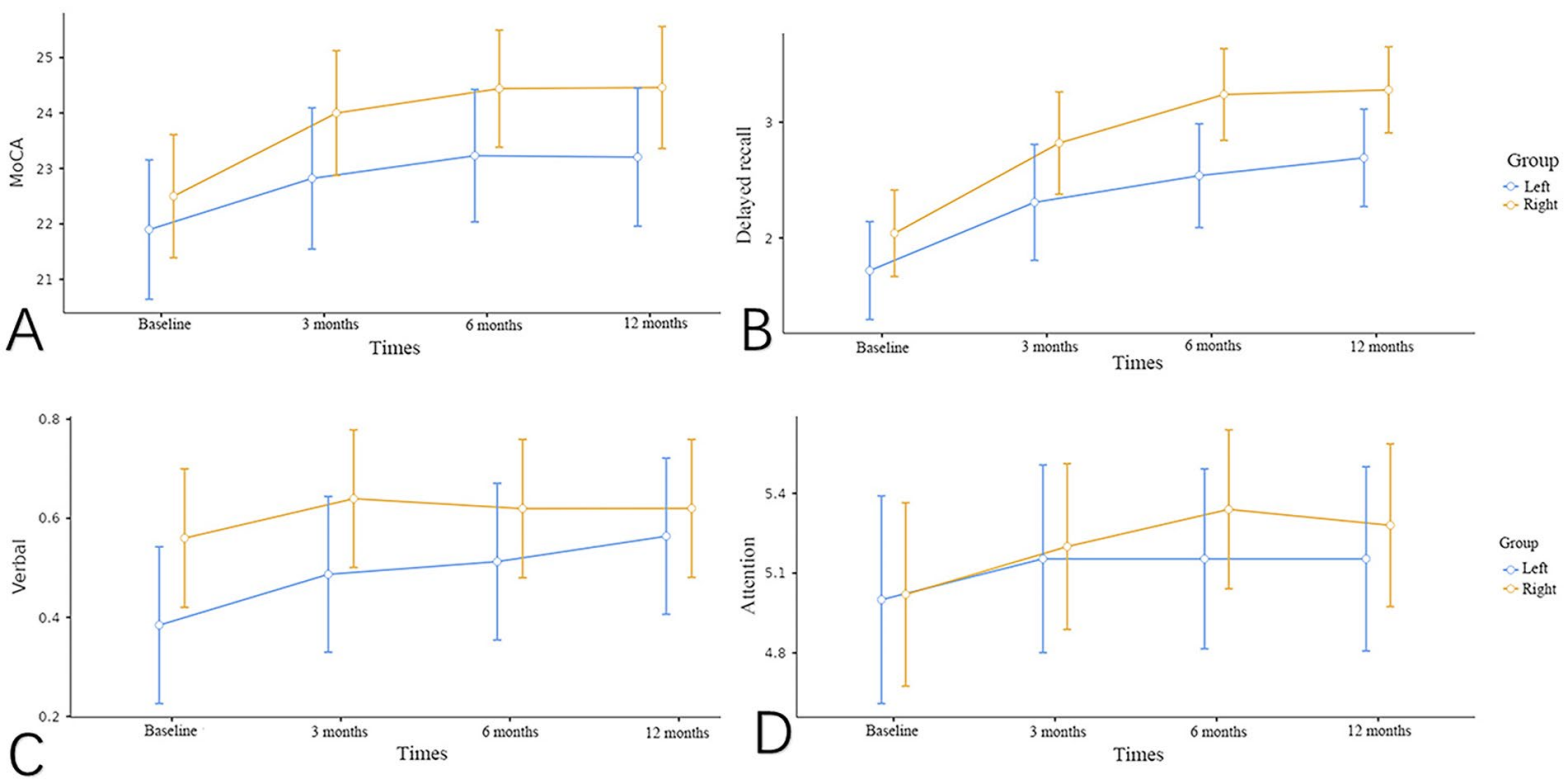
Estimated marginal means for different test items between two groups. **(A)** Shows the MoCA total score in two groups, and **(B)** shows the delayed recall score in two groups, shows an enhancement in cognitive function at 3, 6, and 12 months postoperatively. **(C)** Shows the verbal fluency in two groups; and has improvement at 12 months compared to baseline levels in left group; **(D)** shows the Attention score in two groups; attention showed improvement at 6 months compared to baseline among right group.

The MoCA total scores and subtest scores are summarized in Table 2, and the estimated marginal means of the corresponding subgroups are illustrated in Figure 1.

**Table 2:** Effect of revascularization on cognition among all patients.

Cognition test	Baseline		3 months		6 months		12 months	
	Left	Right	Left	Right	Left	Right	Left	Right
MoCA score	21.90 ± 4.21^a^	22.50 ± 3.73^a^	22.82 ± 4.10^a^	24.00 ± 3.93^a^^,^^b^	23.23 ± 3.84^a^	24.44 ± 3.69^a^^,^^b^	23.21 ± 3.92^a^	24.46 ± 3.91^a^^,^^b^
Alternating trail	0.36 ± 0.49	0.32 ± 0.47	0.33 ± 0.48	0.36 ± 0.48	0.33 ± 0.48	0.40 ± 0.49	0.33 ± 0.48	0.48 ± 0.50
Cube copying	0.64 ± 0.49	0.72 ± 0.45	0.62 ± 0.49	0.80 ± 0.40	0.67 ± 0.48	0.80 ± 0.40	0.67 ± 0.48	0.82 ± 0.39
Clock drawing	2.23 ± 0.84	2.10 ± 0.76	2.23 ± 0.81	2.20 ± 0.70	2.13 ± 0.83	2.28 ± 0.64	2.13 ± 0.86	2.28 ± 0.70
Naming	2.72 ± 0.60	2.78 ± 0.55	2.74 ± 0.59	2.84 ± 0.47	2.77 ± 0.54	2.84 ± 0.47	2.77 ± 0.54	2.86 ± 0.45
Attention	5.00 ± 1.15	5.02 ± 1.29^a^	5.15 ± 1.11	5.20 ± 1.11	5.15 ± 1.11	5.34 ± 1.02^a^	5.15 ± 1.11	5.28 ± 1.07
Sentence repeating	0.97 ± 0.58	1.02 ± 0.71	1.08 ± 0.66	0.96 ± 0.73	1.13 ± 0.66	1.00 ± 0.73	1.15 ± 0.71	1.00 ± 0.73
Verbal fluency	0.38 ± 0.49^a^	0.56 ± 0.50	0.49 ± 0.51	0.64 ± 0.48	0.51 ± 0.51	0.62 ± 0.49	0.56 ± 0.50^a^	0.62 ± 0.49
Abstraction	1.46 ± 0.68	1.34 ± 0.72	1.49 ± 0.68	1.42 ± 0.70	1.54 ± 0.64	1.40 ± 0.70	1.49 ± 0.64	1.38 ± 0.70
Delayed recall	1.72 ± 1.38^a^	2.04 ± 1.29^a^	2.31 ± 1.47^a^	2.82 ± 1.65^a^^,^^b^	2.54 ± 1.33^a^	3.24 ± 1.46^a^^,^^b^	2.69 ± 1.26^a^	3.28 ± 1.37^a^^,^^b^
Orientation	5.62 ± 0.78	5.66 ± 0.56	5.77 ± 0.48	5.76 ± 0.48	5.72 ± 0.60	5.72 ± 0.54	5.74 ± 0.50	5.72 ± 0.54

a *p* < 0.05, comparison of results between postoperative and preoperative.

b *p* < 0.05, comparison of results between postoperative 6 months or 12 months and 3 months.

At baseline, there were no statistically significant differences in the overall MoCA scores or any subtest domains between the two groups. Following carotid revascularization, both groups demonstrated progressive improvement in global cognitive performance over time. MoCA total scores increased significantly at 3, 6, and 12 months postoperatively compared with preoperative levels, suggesting a sustained enhancement in cognitive function (Figure 1A). In patients undergoing left-sided carotid revascularization, delayed recall showed a statistically significant improvement at 3, 6, and 12 months, and verbal fluency improved significantly at 12 months compared with baseline (Figures 1B,C). In contrast, in patients undergoing right-sided revascularization, attention improved significantly at 6 months, and delayed recall improved at 3, 6, and 12 months relative to baseline. Moreover, the improvement in delayed recall at 6 and 12 months remained significant when compared with the 3-month assessment, indicating a cumulative effect over time (Figures 1B,D).

## Discussion

The impact of carotid artery revascularization surgery on cognitive function in patients is currently a topic of debate due to the various factors that influence cognitive changes. Several potential mechanisms have been suggested in current research. (1) Cerebral hypoperfusion (21, 22): studies have shown that mild to moderate carotid artery stenosis does not cause significant cerebral hypoperfusion due to the compensatory mechanisms in the brain. However, severe carotid artery stenosis can lead to significant reduction in cerebral blood flow. Neurological tissues such as the hippocampus and frontal and temporal lobes are highly sensitive to ischemic–hypoxic conditions. When chronic carotid artery stenosis leads to reduced cerebral perfusion, neurons in these regions can generate free radicals due to ischemic–hypoxic stress, leading to oxidative damage and ultimately resulting in cognitive impairment (23). (2) Asymptomatic microembolism: the carotid arteries supply blood to two-thirds of the brain, including regions such as the frontal and temporal lobes, hippocampus, and the limbic system, which are closely associated with cognitive function. Plaque embolization from the carotid artery can block these areas. Microemboli can cause subtle cognitive dysfunction, which might not be readily detected (24, 25). (3) Cerebral white matter lesions (26): white matter lesions disrupt neural connections in the brain and can lead to cognitive impairment. Studies have demonstrated a significant increase in white matter lesions in patients with carotid artery stenosis, which is associated with cognitive dysfunction.

Due to the functional differences between the left and right hemispheres of the human brain, there may be variations in cognitive changes among patients with different locations of carotid artery stenosis. Therefore, the impact of revascularization surgery on functional recovery remains unclear. A study by Johnston et al. (27) found a particularly strong correlation between high-grade stenosis in the left carotid artery and cognitive impairment. However, no such association was observed in patients with right carotid artery stenosis. Specifically, in patients who underwent left carotid artery treatment, there was a significant improvement in cognitive abilities, especially in the domain of language. This improvement may be related to the restoration of blood flow on the same side. However, Bossema et al. (28) used neuropsychological tools sensitive to hemisphere specialization and demonstrated that cognitive changes occur regardless of which side is intervened. They also noted that verbal and language functions improved in patients who underwent left carotid artery surgery, while non-verbal or visuospatial functions were enhanced in those who underwent right-side surgery.

In a subgroup analysis conducted by Scherr et al. (15), they found that cognitive performance improved in measures of global cognition, verbal episodic memory (patients with left-sided CAS) and divided attention (patients with right-sided CAS). Similarly, a study by Japanese researchers observed that the differences in cognitive improvement depended on the intervention site. They conducted a study on 39 patients who underwent CAS and compared the results between the left and right groups. The findings showed that patients with left carotid artery stenosis had improved Verbal intelligence quotient after surgery, while those with right-side stenosis showed improved performance intelligence quotient (29).

Lattanzi et al. also found similar trends in their research (14). They used Coloured Progressive Matrices (CPM) plus Complex Figure Copy Test (CFCT) and phonemic (ph) plus categorical (ca) Verbal Fluency (VF) tests were performed to assess right and left hemisphere cognitive functions, respectively. Patients with left ICA stenosis performed worse on the phonemic and categorical VF tests, and patients with right ICA obtained lower scores on the CPM and CFCT. Cognitive performance was enhanced at 6 months since CEA, and the improvement was related to the cerebral vasomotor reactivity increase. Later on, Lattanzi et al. conducted a study that included 181 patients with severe unilateral carotid artery stenosis and a history of transient ischemic attacks.

At 6 months from CEA, the scores obtained in the cognitive tests exploring the re-vascularized hemisphere’s functions and ipsilateral cerebral hemodynamics were improved (13). The purpose of these tests had to evaluate the cognitive function in the right and left hemisphere. The findings showed that in patients who underwent left carotid artery reconstruction, there was mainly improvement in language-related aspects. In patients who underwent right carotid artery reconstruction, there was mainly improvement in executive functions.

However, not all studies have confirmed the impact of revascularization side on postoperative cognitive function. Baracchini et al. (16) did not observe any effects of the operative side on cognitive test results. They only found that patients with symptomatic carotid artery stenosis showed improved average cognitive test scores after CEA, while no changes were observed in asymptomatic patients. Similarly, Grunwald et al. (18), Yamashita et al. (17) and Turk et al. (30), in their studies on CAS, found no correlation between cognitive outcomes and the side of intervention. The diversity in research results may be caused by multiple confounding factors, such as differences in patient selection variables, sample sizes, choice of cognitive tests, follow-up time, and intervals between postoperative tests. For example, the inclusion of patients with a history of stroke is common in many studies, but the distribution of these patients between the left and right surgical groups may not be equal, or there may be a lack of information in this regard. These confounding factors can potentially influence the research outcomes as the history of stroke itself may be related to cognitive function. Additionally, there are other potential factors, such as age, gender, baseline cognitive status, and comorbidities, which may also have an impact on the results.

MoCA is a simple and comprehensive screening tool for detecting cognitive impairment (31). In this study, we utilized the more sensitive MoCA test scale (32). Additionally, to avoid practice effects in cognitive testing, we employed parallel forms, where each test group had different word lists. Moreover, with the training of professional personnel, we aimed to minimize potential errors. Furthermore, we extended the follow-up time to 12 months and conducted four assessments for the included patients. In our study, we found no significant differences in cognitive scores at baseline, further reducing the impact of preoperative data bias on the results. Our research results are similar to studies conducted by Bossema et al. (28) and Scherr et al. (15). We also found that interventions on different sides can have a certain impact on cognition. Although both groups of patients demonstrated overall improvement in cognition and enhanced delayed recall ability, there were some differences in verbal fluency on the left side and attention on the right side. One possible explanation for the improvement in delayed recall is that carotid artery disease may affect brain functional connectivity beyond the vascular area on the same side, thus influencing cognitive function. Further research is needed to explore the exact mechanisms of how carotid artery surgery affects delayed recall. Therefore, these studies suggest that the impact of carotid revascularization surgery on cognitive function may differ depending on the side and specific cognitive domains. However, further research is still required to better understand the effects of carotid artery revascularization surgery on cognitive function and to elucidate the underlying mechanisms involved.

## Limitations

Despite the advancements made in this study, there are still some limitations. Firstly, the sample size was relatively small, which may have impacted the stability of the results. Future studies could consider increasing the sample size to enhance the reliability and stability of the findings. Secondly, the study did not distinguish between symptomatic and asymptomatic carotid stenosis or between different revascularization procedures (CEA and CAS), which may have affected the results. Furthermore, the study did not explore the relationship between surgical approaches and cognitive function improvement. Future research could focus on investigating the effects of different surgical techniques on patients’ cognitive function. In addition, the MoCA was used as a global screening tool for cognitive evaluation; however, it may lack sufficient sensitivity to detect subtle or domain-specific cognitive changes, particularly those related to hemispheric lateralization. Lastly, brain perfusion MRI/CT and transcranial Doppler monitoring during CAS was not performed in either group, resulting in a lack of objective indicators for microembolism and cerebral perfusion. Follow-up studies should consider incorporating objective measures to further explore their impact.

## Conclusion

Carotid revascularization surgery can improve cognitive function in patients, with differences in cognitive function changes observed between left and right carotid revascularization procedures. In patients undergoing left carotid revascularization surgery, improvements were observed in verbal fluency and delayed recall, while in patients undergoing right carotid revascularization surgery, attention and delayed recall showed significant improvements.

## Data Availability

The raw data supporting the conclusions of this article will be made available by the authors, without undue reservation.
